# Re-Designed Recombinant Hepatitis B Virus Vectors Enable Efficient Delivery of Versatile Cargo Genes to Hepatocytes with Improved Safety

**DOI:** 10.3390/v8050129

**Published:** 2016-05-10

**Authors:** Weiya Bai, Xiaoxian Cui, Ruidong Chen, Shuai Tao, Ran Hong, Jiming Zhang, Junqi Zhang, Yongxiang Wang, Youhua Xie, Jing Liu

**Affiliations:** 1 Key Laboratory of Medical Molecular Virology (MOH & MOE) and Institutes of Biomedical Sciences, School of Basic Medical Sciences, Shanghai Medical College, Fudan University, Shanghai 200032, China; bwy.1989@163.com (W.B.); cuixiaoxian9@hotmail.com (X.C.); chenruidong1000@163.com (R.C.); 13211010047@fudan.edu.cn (S.T.); mol725@163.com (R.H.); junqizhang@fudan.edu.cn (J.Z.); yxwang712@yahoo.com (Y.W.); 2 Department of Infectious Diseases, Huashan Hospital, Fudan University, Shanghai 200040, China; jmzhang@fudan.edu.cn

**Keywords:** HBV, gene therapy, viral vector, hepatocyte

## Abstract

Hepatitis B virus (HBV) takes humans as its sole natural host, and productive infection *in vivo* is restricted exclusively to hepatocytes in the liver. Consequently, HBV-derived viral vectors are attractive candidates for liver-targeting gene therapies. Previously, we developed a novel recombinant HBV vector, designated 5c3c, from a highly replicative clinical isolate. 5c3c was demonstrated to be capable of efficiently delivering protein or RNA expression into infected primary tupaia hepatocytes (PTH), but the design of 5c3c imposes stringent restrictions on inserted sequences, which have limited its wider adoption. In this work, we addressed issues with 5c3c by re-designing the insertion strategy. The resultant vector, designated 5dCG, was more replicative than parental 5c3c, imposed no specific restrictions on inserted sequences, and allowed insertion of a variety of cargo genes without significant loss of replication efficiency. 5dCG-based recombinant HBV effectively delivered protein and RNA expression into infected PTH. Furthermore, due to the loss of functional core ORF, 5dCG vectors depend on co-infecting wild type HBV for replication and efficient expression of cargo genes. Development of the improved 5dCG vector makes wider applications of recombinant HBV possible, while dependence on co-infecting wild type HBV results in improved safety for certain *in vivo* applications.

## 1. Introduction

Humans are the only known natural host of hepatitis B virus (HBV) and so far, the only cell type consistently shown to support productive HBV infection cycle has been hepatocytes [1]. Such highly restricted host and tissue tropism of HBV makes it ideal as a vector for liver-targeting gene delivery *in vivo*. Other viral vectors, including adenovirus and adeno-associated virus, lack such tissue specificity, and infection of human cell types might cause unwanted effects that are difficult to test *in vitro* or in animal models [2]. For *in vitro* applications, recombinant HBV vectors expressing easily detectable and quantifiable reporters are powerful tools for addressing important issues such as HBV infection and replication mechanisms.

**Figure 1:**
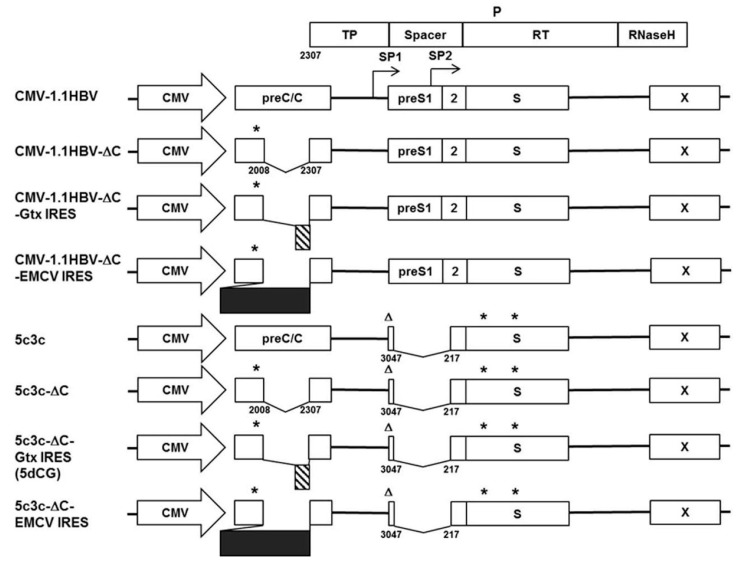
Recombinant HBV vector design scheme. Constructs used in this study are schematically depicted and aligned to wild type HBV construct CMV-1.1HBV to illustrate positions and relative lengths of deletions and artificially introduced sequence units. Wild type HBV polymerase ORF that overlaps with preC/C, preS1/preS2/S and X ORFs is depicted above CMV-1.1HBV. Asterisks denote premature termination codons in preC/C and S ORF. Triangles denote ATG to ACG mutation of preS1 start codon that does not alter polymerase amino acid sequence. Artificial Gtx and EMCV IRES sequences are represented as patterned and solid boxes, respectively. CMV, cytomegalovirus promoter. P, HBV polymerase. TP, terminal protein domain. RT, reverse transcriptase domain. Numbers indicate nucleotide positions on the viral genome.

The HBV genome is highly compact and extremely economical in terms of space usage. Four overlapping ORFs (preC/C, P, preS1/preS2/S, X) (Figure 1) encompass the entire genome, which also contains multiple *cis*-acting elements essential for various steps of the viral life cycle [1,3]. Partially double-stranded relaxed circular DNA (rcDNA) genomes in mature HBV virions are converted into covalently closed circular DNA (cccDNA) upon infection, which then serves as intranuclear transcription template for all viral RNA species [1,3]. Viral polymerase is translated from pregenomic RNA (pgRNA) and binds the latter *in cis* to initiate reverse transcription, which is the first step in genome replication [1,3,4]. Packaging of the pgRNA/polymerase complex by viral core proteins, also translated from pgRNA, is required for subsequent steps of replication and eventual formation of rcDNA. Mature capsids are coated by host-derived membranes containing viral large, middle and small (L/M/S) envelope proteins, encoded by the preS1/preS2/S ORF, and bud into ER lumen to produce progeny virions [1,3].

The compact and overlapping genome organization, combined with a fairly strict limit on genome size imposed by capsids and polymerase [5,6], makes engineering HBV for vector use complicated [5,7,8]. In addition, although *trans-*complemented polymerase does support a certain level of replication, recombinant pgRNA encoding functional polymerase that acts *in cis* usually replicates with much better efficiency [4,8]. Previously, we circumvented these difficulties by modifying a highly replicative clinical isolate of HBV with a large in-frame deletion in preS/polymerase spacer region into a recombinant HBV vector system designated 5c3c (Figure 1). 5c3c contains a maximized deletion in polymerase spacer region, where cargo gene is inserted, and replicates more efficiently than wild type HBV [9]. Cargo sequences must be carefully selected or synonymously mutated to avoid introducing stop codons in the overlapping polymerase ORF. 5c3c-based constructs displayed replication efficiencies comparable to wild type HBV and produced mature virions efficiently when *trans-*complemented with envelope proteins in transfected cells. Furthermore, in an *in vitro* model of HBV infection using primary tupaia hepatocytes (PTH), these 5c3c-based recombinant virions were demonstrated to be infective and deliver expression of cargo protein or RNA genes upon infection [9].

Despite such demonstrable usability, the 5c3c vector system has limitations that hinder its wider adoption and application. Firstly, the restriction on insertion sequence properties limits possible cargo gene choices. Even when inserted sequences do not introduce premature stop codons into polymerase ORF, unrelated amino acid sequences are inevitably introduced into the polymerase spacer, which may affect polymerase activity and, consequently, replication efficiency. High replication efficiency, however, is a key feature distinguishing 5c3c from most previous recombinant HBV attempts [5,7,8]. Additionally, although mature virion production by 5c3c vectors requires *trans-*complemented envelope proteins, replication of 5c3c-based recombinant genomes is self-sufficient and does not rely on co-existing wild type HBV functions [9]. This may be considered a safety issue in certain *in vivo* applications where persistent activity of recombinant HBV is undesirable or even harmful.

In this work, we addressed the above issues with 5c3c by redesigning the cargo gene insertion strategy and engineered a significantly improved derivative vector designated 5dCG. 5dCG is not affected by the insertion sequence restrictions of 5c3c, while retaining a relatively high replication efficiency. Furthermore, cargo gene expression, genome replication and progeny virus production of 5dCG is dependent on *trans-*complemented core and envelope proteins from co-infecting wild type HBV. Infectivity and cargo gene expression of 5dCG-based recombinant HBV were demonstrated using PTH and properties of 5c3c and 5dCG are comparatively discussed in the context of potential applications.

## 2. Materials and Methods

### 2.1. Plasmids

Construction of the recombinant HBV vector 5c3c has been reported previously [9]. The recombinant 5c3c genome contains an in-frame deletion (nucleotides 3047-3215/1-217) in the polymerase spacer region (Figure 1) created by maximizing a naturally occurring deletion in the parental clinical isolate (GenBank accession number FJ518810), where cargo sequences could be inserted. In the overlapping preS1/preS2/S ORF, preS1 start codon was mutated from ATG to ACG, which does not change polymerase amino acid at this position. Additionally, S ORF contains 2 premature stop codons inherited from the parental clinical isolate. The genome was cloned into pHY106 vector [10] downstream of the cytomegalovirus (CMV) promoter to create 5c3c. Wild type HBV genome was amplified from p1.2-HBV containing 1.2 copy of HBV genome [11] and similarly cloned into pHY106 to create CMV-1.1HBV. Envelope-expressing helper plasmid pLMS encoding wild type envelope proteins was constructed by deleting sequences upstream of the Sp1 promoter from p1.2-HBV. Core-expressing helper plasmid pC was constructed by amplifying core-encoding sequences from 5c3c and cloning into pCDH-CMV-MCS vector (System Biosciences) downstream of CMV promoter.

To test alternative cargo gene insertion sites, 298 bp (nucleotides 2009–2306) of core coding sequences immediately upstream of polymerase start codon were deleted from 5c3c and CMV-1.1HBV to create 5c3c-ΔC and CMV-1.1HBV-ΔC, respectively, using KOD-Plus-Mutagenesis Kit (TOYOBO, Osaka, Japan). A 21 bp linker (TAACC*CTGCAG*GCTC*GCTAGC*) containing a termination codon (underlined) that terminates the preceding preC/C ORF as well as *Pst*I and *Nhe*I recognition sites (underlined and italicized) was introduced in the process to facilitate subsequent insertion of cargo sequences. The 582 bp encephalomyocarditis virus (EMCV) internal ribosomal entry site (IRES) and an 82 bp artificial IRES based on minimal IRES sequences in cellular Gtx gene mRNA, designated (Gtx133-141)2(SII)1β in reference [12], were chemically synthesized and inserted into 5c3c-ΔC and CMV-1.1HBV-ΔC between the linker and polymerase start codon. The resultant constructs were designated 5c3c-ΔC-EMCV-IRES, 5c3c-ΔC-Gtx-IRES and CMV-1.1HBV-ΔC-EMCV-IRES, CMV-1.1HBV-ΔC-Gtx-IRES, respectively (Figure 1). For brevity, ‘IRES’ in construct names is sometimes omitted and the vector shown to possess the best properties, 5c3c-ΔC-Gtx-IRES, was abridged as 5dCG and used for subsequent recombinant virus construction. Nucleotide sequences of 5dCG surrounding the cargo gene insertion site are shown in Figure S1.

For insertion of protein-encoding genes into 5dCG, sequences encoding zeocin resistance (ZeoR), NanoLuc (NLuc) (Promega, Madison, WI, USA), secreted form of NanoLuc (sNLuc) (Promega), DsRed and EGFP proteins were amplified by PCR from corresponding expression plasmids and inserted into 5dCG using the linker upstream artificial IRES.

For insertion of short hairpin RNA expression cassettes into 5dCG, sequences containing H1 promoter and downstream shRNA-encoding sequences were amplified from previously reported and verified pSuper based plasmids [9]. Briefly, Xi targets 5′-CCAG(A)GTC(G)TTG(A)CCCAAGGTCTTACAT-3′ sequences in X ORF, while Ci targets 5′-GATCTC(T)AAT(C)CTC(T)GGG(A)AAT(C)CTCA-3′ sequences in C ORF. The underlined nucleotides were mutated to parenthesized nucleotides to create Xi-resistant Xm and Ci-resistant Cm sequences, respectively.

### 2.2. Cell Culture, Transfection, and HBV Nucleic Acid Analysis

Huh-7 cells were cultured at 37 °C and 5% CO2 in Dulbecco’s modified Eagle medium (DMEM) containing 2 mM L-glutamine, 100 U/mL penicillin, 100 μg/mL streptomycin supplemented with 10% fetal bovine serum (all reagents were from Invitrogen, Carlsbad, CA, USA). Transfections were performed at 80 to 90% confluence using plasmid DNA and polyethylenimine (Sigma, St. Louis, MO, USA) at 1:2 ratio. Empty vectors were included whenever necessary to ensure equal amounts of DNA were used in parallel transfections, while control plasmid encoding firefly luciferase reporter (Promega) was co-transfected for transfection efficiency normalization when appropriate. Extraction of intracellular capsid-associated DNA and extracellular capsid- and virion-associated DNA was performed as previously described [9]. Extracellular Dane particles were enriched by immunoprecipitation using anti-preS1 monoclonal antibody 125E11 (Alpha Inc., Shanghai, China) as previously described [9] and viral DNA was extracted similarly as above. To demonstrate lack of rcDNA formation by 5c3c-ΔC in the absence of *trans*-complemented core, total cellular DNA was extracted using QIAamp DNA Mini Kit (Qiagen, Hilden, Germany) and digested with *Dpn*I (New England BioLabs, Ipswich, MA, USA) to fragment transfected plasmid DNA. Extracted HBV DNA was analyzed via Southern blot using a digoxigenin labeled HBV-specific probe encompassing nucleotides 96 to 1509 of the wild-type HBV genome prepared by using PCR DIG Probe Synthesis Kit (Roche, Mannheim, Germany). Chemiluminescent detection of hybridized probes was performed using DIG High Prime DNA Labeling and Detection Starter Kit II (Roche).

### 2.3. CsCl Density Gradient Centrifugation

Transfection supernatants, or HBV-infected patient serum, were concentrated using Amicon Ultra-15 (Millipore, Boston, MA, USA) and subjected to CsCl density gradient (1.1 to 1.4 g/cm^3^) centrifugation at 38,000 rpm for 20 h using SW41 rotor on Beckman L80 (Beckman Coulter, Brea, CA, USA). Fractions were assayed for HBsAg using Abbott Architect system, and HBV DNA was extracted and analyzed using Southern blot as described above.

### 2.4. Infection of Primary Tupaia Hepatocytes

PTH were prepared following a two-step perfusion protocol as previously described [13]. Freshly prepared PTH were seeded into collagen-coated 48-well plates (BD Biosciences, Bedford, MA, USA) at 1 × 10^5^ cells per well and maintained in Hepato-Stim Hepatocyte Defined medium (BD Biosciences). For infection assays, Amicon Ultra-15-concentrated transfection supernatants containing about 1 × 10^7^ HBV genome equivalents (geq), quantitated using a real-time PCR HBV DNA detection kit (Qiagen), were added to each well at 16 h post seeding. After incubation at 37 °C overnight, cells were washed 5 times with PBS and cultured at 37 °C with media changed every day or every other day. For coinfection assays, wild-type HBV virions prepared from patient sera and recombinant HBV particles prepared from transfection supernatants were used at a 1:3 ratio with 1 × 10^7^ geq wild type HBV and 3 × 10^7^ geq recombinant HBV per well.

### 2.5. NanoLuc Activity Assay

Culture supernatants of transfected Huh-7 cells or infected PTH were assayed for levels of secreted NanoLuc using Nano-Glo Luciferase Assay System (Promega) following manufacturer’s instructions. For analysis of intracellular NanoLuc activity, cells were washed and diluted in PBS, and then assayed similarly as culture supernatants.

### 2.6. Immunofluorescence

Fifteen days post infection, PTH were fixed and analyzed using anti-HBcAg (1:700; Dako, Carpinteria, CA, USA) as described previously [9]. Images were captured using AMG EVOS Fluorescence Microscope (AMG, Mill Creek, WA, USA).

## 3. Results

### 3.1. Improving Recombinant HBV Vector 5c3c by Redesigning the Insertion Strategy

Previously, we established a highly replicative recombinant HBV vector designated 5c3c. Compared to wild type HBV, 5c3c possesses a 384bp deletion in preS1/polymerase spacer region (nucleotides 3047-3215/1-217), where carefully selected or engineered cargo sequences could be inserted that must not prematurely terminate the overlapping polymerase ORF so that replication competence could be retained. This requirement severely limited possible choices of cargo sequences of 5c3c. 5c3c maximally tolerates ~678 bp insertions and beyond that limit, replication competence deteriorated to unacceptable levels [9]. Furthermore, 5c3c vectors do not encode HBV envelopment proteins, but they do encode both functional core and polymerase proteins. In infected cells, recombinant 5c3c viruses would be capable of replicating their genomes by themselves, while production of mature enveloped virions would require envelope proteins provided by co-infecting wild type HBV. This might be desirable for applications requiring sustained high expression of cargo genes as a larger cccDNA pool could be achieved with or without wild type co-infection [14,15]. For certain other *in vivo* applications, however, such self-sufficient replication and expansion of delivery vector could be considered an unnecessary risk.

**Figure 2:**
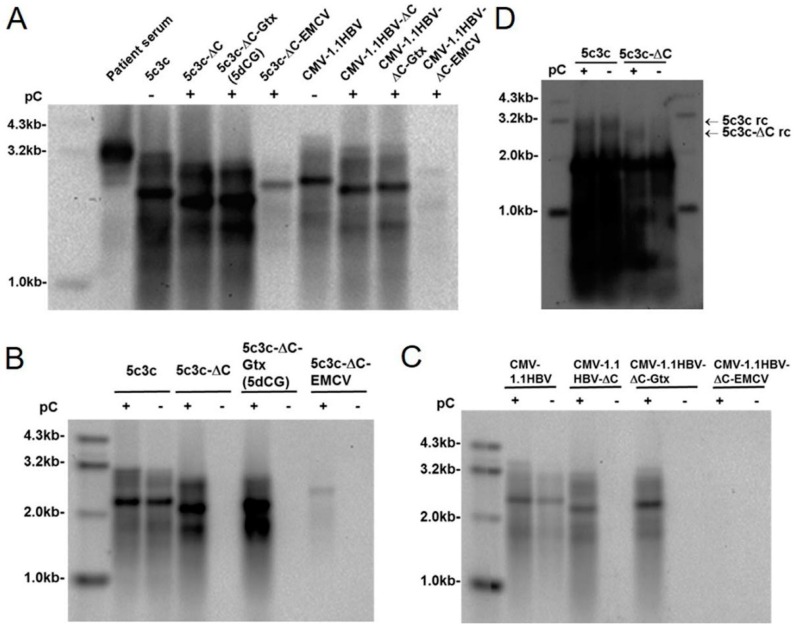
Replication of recombinant HBV vectors in Huh-7 cells. (**A**) Southern blot analysis of replication of 5c3c and wild type HBV constructs with deletions in C ORF. Intracellular capsid-associated HBV DNA was prepared from Huh-7 cells transfected with indicated plasmids. Viral DNA was also prepared from HBV-infected patient serum to demonstrate the position of mature full-length wild type rcDNA; (**B**,**C**) Southern blot analysis of replication of 5c3c (**B**) and wild type HBV (**C**) constructs with deletions in C ORF in the presence or absence of *trans-*complemented HBV core. Intracellular capsid-associated HBV DNA was prepared from Huh-7 cells transfected with indicated plasmids; (**D**) Deletion in C ORF of 5c3c abolished rcDNA formation. Total DNA was extracted from Huh-7 cells transfected with indicated plasmids and analyzed in Southern blot. Positions of mature rcDNA species are indicated. Transfection efficiency was normalized using relative luciferase activities expressed by co-transfected firefly luciferase reporter measured using Luciferase Assay System (Promega).

In an attempt to solve these issues, alternative insertion strategies were tested for 5c3c. Since polymerase ORF has to be preserved to ensure high replication competence, whereas X ORF and the N-terminal part of preC/C ORF contain various *cis*-acting elements required for genome replication and packaging (core promoter, DR1, DR2, epsilon signal, *etc.*) [1,3], the only option other than preS/polymerase spacer region was the central part of preC/C ORF that does not overlap with polymerase ORF (Figure 1). Previously, this region has been targeted in recombinant HBV studies with limited inconclusive results [16,17]. We deleted 298 bp of this region (nucleotides 2009–2306) in 5c3c and inserted a short synthetic linker containing both a stop codon to terminate core ORF prematurely and restriction enzyme recognition sites to ease subsequent insertions. The resultant construct was named 5c3c-ΔC (Figure 1). In the presence of *trans-*complemented core, 5c3c-ΔC demonstrated slightly higher replication efficiency compared to parental 5c3c (Figure 2A,B), probably due to its further reduced genome size. More importantly, loss of functional core ORF rendered 5c3c-ΔC incapable of genome replication by itself: no rcDNA could be detected when total cellular DNA from cells transfected with 5c3c-ΔC alone was analyzed (Figure 2D), whereas in cells co-transfected with 5c3c-ΔC and core-expressing plasmid pC, 5c3c-ΔC rcDNA was easily demonstrated in both intracellular capsid-associated DNA and total cellular DNA (Figure 2B,D). In contrast, the parental core-encoding 5c3c efficiently produces rcDNA regardless of core co-transfection (Figure 2B,D). This is in agreement with the current understanding of HBV replication mechanisms, which views packaging of pre-gnomic RNA-polymerase complex by capsid as a prerequisite for productive rcDNA synthesis.

### 3.2. Short Artificial IRES Inserted Upstream of Polymerase ORF Improves 5c3c-ΔC Replication

To ensure that polymerase translation is minimally affected by insertion of cargo sequences, IRES sequences were then inserted immediately upstream of polymerase start codon in 5c3c-ΔC to isolate polymerase translation from upstream sequences. Insertion of the 582 bp EMCV IRES into 5c3c-ΔC (5c3c-ΔC-EMCV) resulted in a recombinant HBV genome of 3150 bp, slightly shorter than wild type HBV (3215bp). However, 5c3c-ΔC-EMCV replicated very poorly compared to 5c3c-ΔC, 5c3c and wild type HBV (CMV-1.1HBV) (Figure 2A,B). Alternatively, an 82 bp artificial IRES was synthesized based on a 9 nt minimal IRES unit identified in the Gtx homeodomain protein pre-mRNA [12] and was designated Gtx IRES. 5c3c-ΔC harboring Gtx IRES upstream of polymerase ORF (5c3c-ΔC-Gtx, abbreviated as 5dCG, Figure S1) demonstrated improved replication efficiency over the parental 5c3c-ΔC (Figure 2A,B). Compared to EMCV IRES, the much shorter Gtx IRES not only provides better replication competence, but also would allow insertion of much longer cargo sequences given the same genome size limit of HBV [5,6].

Similar results were also obtained using wild type HBV (CMV-1.1HBV) instead of 5c3c as starting vector: CMV-1.1HBV-ΔC requires *trans-*complemented core expression to fulfill replication; insertion of EMCV IRES in CMV-1.1HBV-ΔC upstream of polymerase nearly obliterated replication competence, while insertion of Gtx IRES allowed the recombinant genome to replicate as efficiently as CMV-1.1HBV-ΔC or CMV-1.1HBV (Figure 2A,C). Generally, 5c3c-based constructs displayed more efficient replication compared to corresponding constructs based on wild type HBV containing full-length polymerase (Figure 2A–C). This is in agreement with our previous results and highlighted again the significant benefit for replication provided by the deletion in polymerase spacer in 5c3c [9]. 5dCG demonstrated the highest replication competence among all tested constructs and was selected as the recombinant HBV vector for subsequent work.

### 3.3. 5dCG Allows Versatile Choice of Cargo Sequences

**Figure 3:**
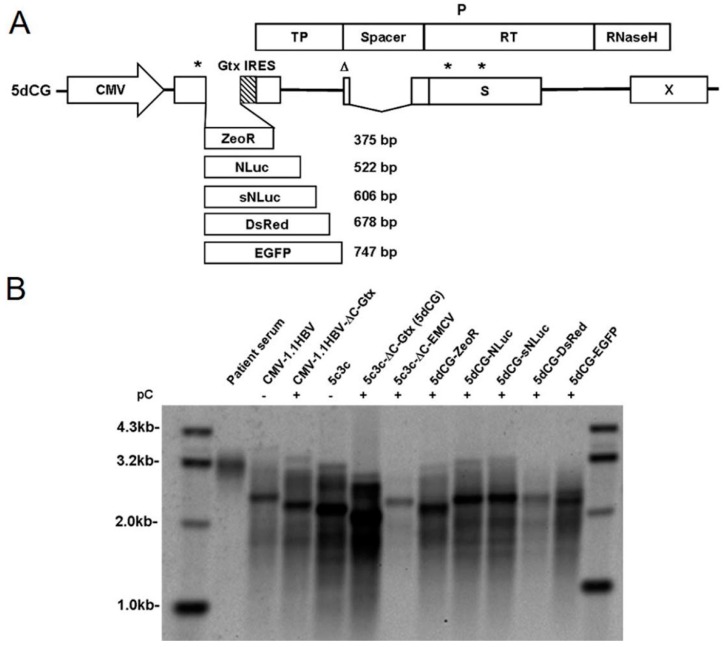
Recombinant HBV vector 5dCG allows versatile choice of cargo genes. (**A**) Schematic representation of 5dCG vector and multiple cargo genes tested. Cargo genes are represented as open boxes approximately drawn to scale and the actual lengths indicated to the right. Wild type HBV polymerase ORF that overlaps with preC/C, preS1/preS2/S and X ORFs is also depicted. Asterisks denote premature termination codons in preC/C and S ORF. Triangles denote ATG to ACG mutation of preS1 start codon that does not alter polymerase amino acid sequence. Artificial Gtx IRES sequences are represented as a patterned box. CMV, cytomegalovirus promoter. P, HBV polymerase. TP, terminal protein domain. RT, reverse transcriptase domain; (**B**) Southern blot analysis of replication of 5dCG vector harboring different cargo genes. Intracellular capsid-associated HBV DNA was prepared from Huh-7 cells transfected with indicated plasmids. Viral DNA was also prepared from HBV-infected patient serum to demonstrate the position of mature full-length wild type rcDNA. Transfection efficiency was normalized using relative luciferase activities expressed by co-transfected firefly luciferase reporter measured using the Luciferase Assay System (Promega).

To test the cargo-carrying capacity of 5dCG, sequences of varying lengths that encode 5 commonly used protein reporters, ZeoR, NLuc, sNLuc, DsRed and EGFP, were amplified from corresponding expression plasmids and inserted, without sequence modification, into 5dCG upstream of Gtx IRES (Figure 3A). The inserted ORFs contained their own start and stop codons, and ranged in size from 375 to 747 bp. In the presence of a *trans-*complemented core, 5dCG constructs carrying up to 606 bp insertion (5dCG-sNLuc) displayed replication efficiencies better than wild type HBV, but inferior to 5c3c or 5c3c-ΔC (Figure 3B). Insertion of the 678 bp DsRed-encoding sequences resulted in decreased replication compared to wild type, while insertion of 747 bp EGFP-encoding sequences did not cause a marked decrease of replication intermediates (Figure 3B). These results demonstrated that both length and other properties of the inserted sequences could affect replication competence and 5dCG potentially tolerates insertions of up to ~750 bp.

**Figure 4:**
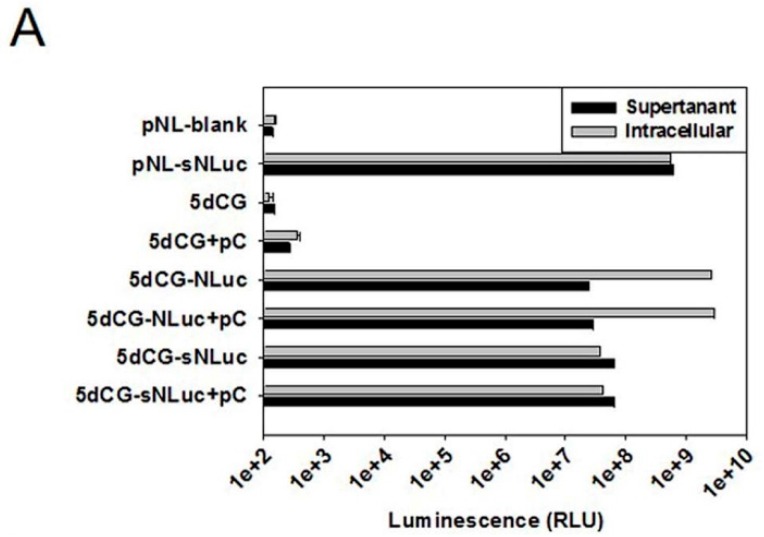

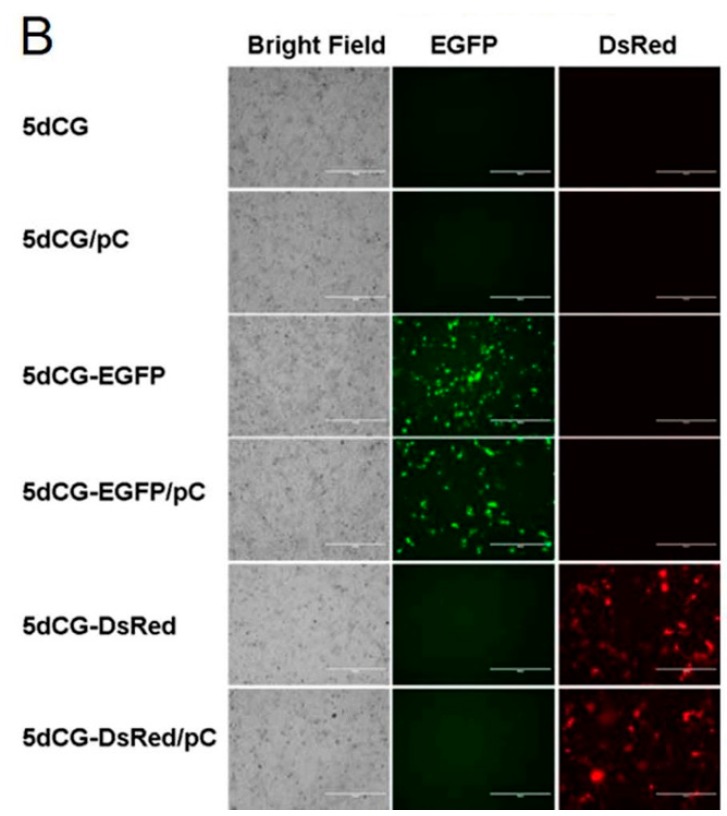
Cargo gene expression from 5dCG-based recombinant HBV plasmids. (**A**) Assay of intracellular and supernatant NanoLuc activity. Huh-7 cells were transfected with indicated plasmids and culture media and cells were harvested 3 days post transfection. Both harvested cells and supernatants were diluted 1200-fold in PBS prior to analysis using Nano-Glo Luciferase Assay System (Promega). pNL-sNLuc and pNL are over-expression plasmid of sNLuc purchased from Promega and its corresponding empty vector, and were used as positive and negative controls, respectively. Transfection efficiency was normalized using relative luciferase activities expressed by co-transfected firefly luciferase reporter measured using Luciferase Assay System (Promega); (**B**) Fluorescent imaging of EGFP and DsRed expression. Huh-7 cells transfected with indicated plasmids were imaged using AMG EVOS Fluorescence Microscope.

When expression of bioluminescent (Figure 4A) and fluorescent (Figure 4B) cargo genes from 5dCG-based constructs was tested in transfected Huh-7 cells, all tested constructs displayed easily detectable reporter expression, with or without core co-transfection, demonstrating that the inserted ORFs were fully functional. Replication efficiency did not seem to have any significant impact on expression of inserted genes, which could be expected as plasmid-driven transcription most likely far exceeds cccDNA-driven transcription in such experiments.

**Figure 5:**
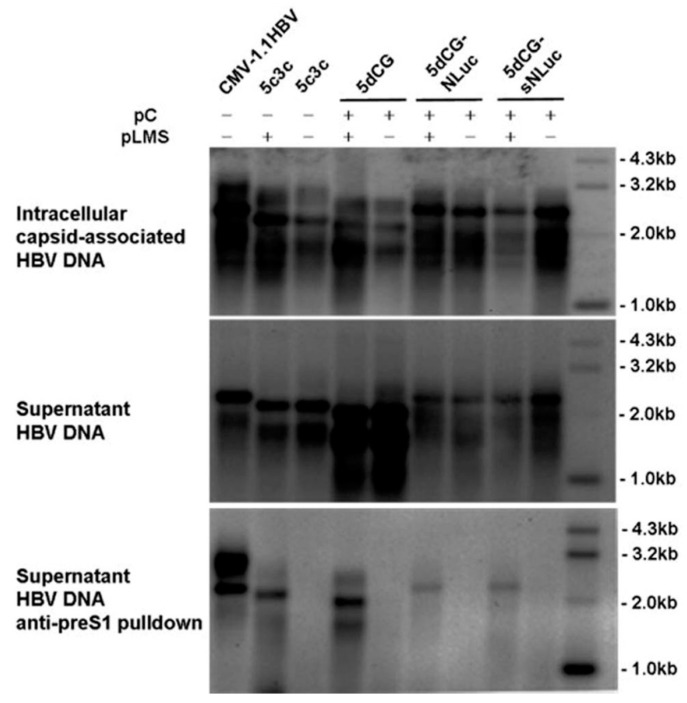
Virion production by 5dCG-based recombinant HBV vectors. HBV DNA in intracellular capsids (**top**), extracellular capsids and virions (**middle**) and extracellular virions (**bottom**) was extracted from Huh-7 cells transfected with indicated plasmids. Virions in supernatants were separated from unenveloped capsids by immunoprecipitation using anti-preS1 monoclonal antibody. In order to provide reference positions of wild type HBV rcDNA, CMV-1.1HBV was transfected at 3.3 fold amount of 5c3c or 5dCG vectors. Transfection efficiency was normalized using relative luciferase activities expressed by a co-transfected firefly luciferase reporter measured using the Luciferase Assay System (Promega).

The ability of 5dCG and derivative recombinant HBV constructs to produce and secrete mature virions was then tested by transfecting Huh-7 cells with the recombinant constructs and core-expressing plasmid (pC), with or without an envelope-expressing plasmid (pLMS). As shown in Figure 5, only in the presence of *trans-*complemented HBV envelope proteins could mature recombinant 5c3c or 5dCG virions be detected in transfection supernatants, whereas intracellular replication could be detected with or without envelope proteins present. When transfection supernatants were subjected to density-gradient ultracentrifuge analysis, both 5dCG- and 5dCG-sNLuc-derived recombinant HBV virions displayed buoyant densities identical to wild type HBV virions from patient serum and wild type HBV transfection supernatants (Figure S2). Non-enveloped DNA-containing core particles were also identified in transfection supernatants, but not in patient serum, which is consistent with previous reports [9,18,19].

Taken together, these data indicated that, compared to parental 5c3c, 5dCG offers much freer choice of cargo genes and tolerates slightly longer insertions, without significant losses in genome replication and virion production efficiency. In the meantime, self-sufficient replication of 5dCG vectors is obliterated, providing freater safety for use cases where long-lasting expansion of vectors and persistent high-level expression of cargo genes are not desirable or necessary.

### 3.4. 5dCG-sNL Allows Non-Invasive Quantitative Detection of Recombinant HBV Infection

The cargo gene in 5dCG-sNL is a secreted form of novel luciferase NanoLuc and the protein product could be quantitatively detected with a dynamic range of multiple orders of magnitude in culture supernatant of transfected or infected cells, as well as in serum of infected animals and patients. This characteristic would be extremely useful for monitoring recombinant virus infection and persistence *in vivo* in preclinical and clinical studies. As a demonstration of such an application, we infected primary tupaia hepatocytes (PTH) with 5dCG-sNL-derived recombinant virus.

**Figure 6:**
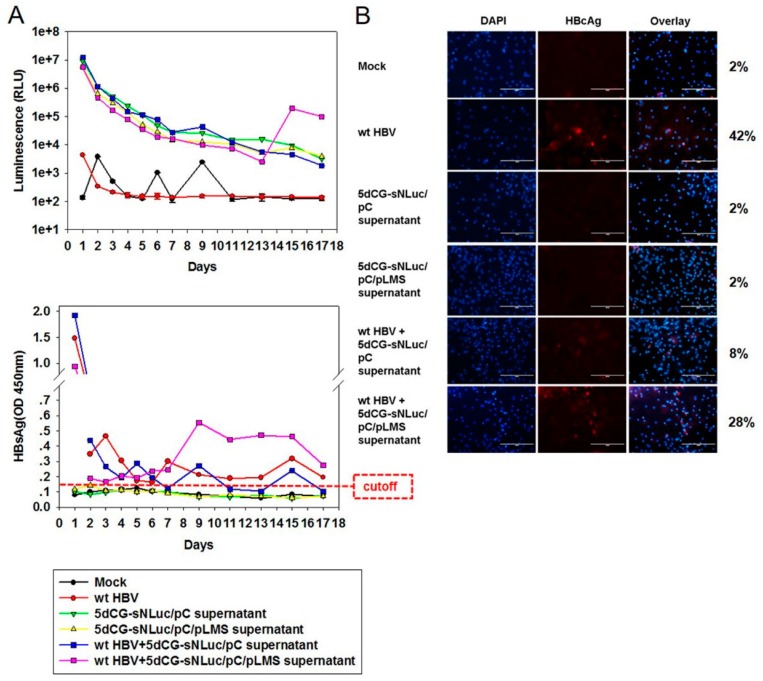
Infection of primary tupaia hepatocytes (PTH) by 5dCG-sNLuc. (**A**) Analysis of NanoLuc (**top**) and HBsAg (**bottom**) in infected PTH supernatants. Freshly prepared PTH were infected with wild type HBV and/or transfected Huh-7 cell supernatants as indicated. Culture media were changed and collected at indicated time points, and assayed for NanoLuc activity and HBsAg; (**B**) Immunofluorescent analysis of HBcAg expression in infected PTH. Infected cells were fixed at day 17 post infection and intracellular HBcAg was detected using anti-HBcAg (Dako). Percentages of HBcAg-positive cells were calculated from ten randomly selected views and listed on the right. Cell nuclei were stained using DAPI. Scale bars, 200 µm.

As shown in Figure 6A (top), due to the presence of large amounts of secreted NanoLuc (sNL) in supernatants of cells transfected or co-transfected with 5dCG-sNL (Figure 4), inoculation of PTH with such supernatants introduced a high background of NanoLuc activity that gradually decreased day by day. PTH infected with 5dCG-sNL virus alone did not secrete marked higher-than-background levels of sNL by day 17 post infection (p.i.), whereas PTH co-infected with wild type HBV and 5dCG-sNL viruses exhibited a surge in secreted sNL activity of nearly 2 logs at day 15 p.i., which then dropped slightly at day 17 p.i., but was still higher than mono-infection by 5dCG-sNL virus as well as other controls by at least 2 logs (Figure 6A, top). No such surge in sNL secretion was observed if 5dCG-sNL transfection supernatants with only *trans*-complemented core, but not envelope proteins, were used for inoculation, regardless of wild type HBV co-infection. In other words, enveloped mature 5dCG-sNL virions (Figure 5) were required for infection and sNL expression.

Secreted HBsAg and intracellular HBcAg were also analyzed to monitor wild type HBV infection and expression. Similar to sNL, inoculation with wild type HBV transfection supernatants resulted in a gradually decreasing background of HBsAg in PTH culture media due to the introduction of large amounts of subviral particles (Figure 6A, bottom). By day 7–9 p.i., however, PTH infected or co-infected with wild type HBV started exhibiting increased HBsAg secretion, which persisted with some fluctuations till day 17 p.i. (Figure 6A, bottom). Correspondingly, immunofluorescent staining for intracellular HBcAg at day 17 p.i. only detected HBcAg expression in PTH infected or co-infected with wild type HBV (Figure 6B). Since 5dCG-sNL does not encode either core or envelope proteins, no HBsAg or HBcAg was detected in 5dCG-sNL mono-infection.

These data demonstrated that enveloped, mature 5dCG-sNL virus was capable of infecting PTH, but marked expression of the cargo gene was only detectable in the presence of wild type HBV co-infection, most likely as a result of lack of self-sufficient replication by 5dCG vectors and *trans-*complementation by co-infecting wild type HBV virus that rescues 5dCG replication (Figure 2). This might also explain why the start of sNL secretion lagged by about 5 days behind start of HBsAg secretion in 5dCG-sNL virus and wild type HBV co-infection (Figure 6A). Compared to self-replicating recombinant HBV vectors like the parental 5c3c, such wild type HBV-dependent expression of cargo gene of 5dCG makes it particularly suitable for HBV-targeting therapeutic applications *in vivo*.

### 3.5. 5dCG Expressing HBV-Targeting RNA Interference Inhibited Co-Infecting Wild Type HBV

**Figure 7:**
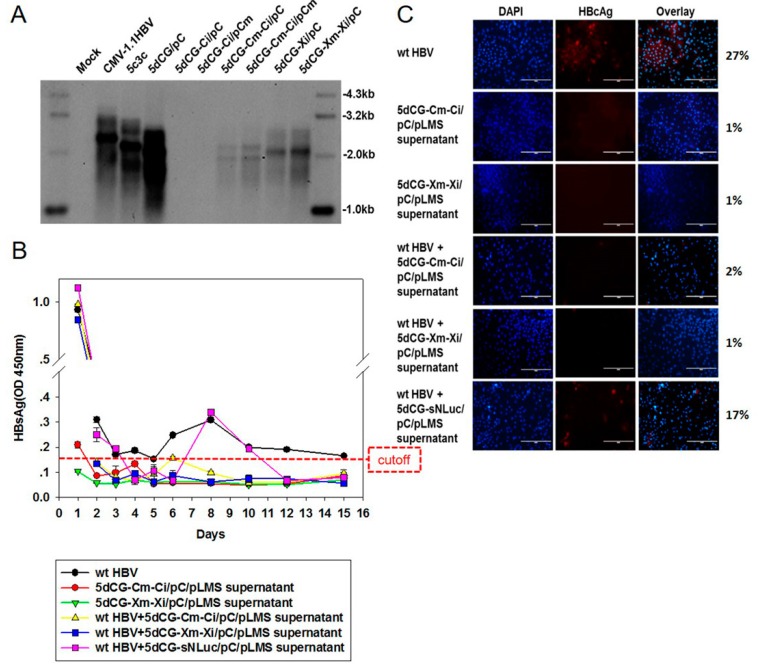
5dCG-delivered RNA interference inhibited co-infecting wild type HBV. (**A**) Southern blot analysis of replication of 5dCG constructs harboring HBV-targeting siRNA precursors. Intracellular capsid-associated HBV DNA was prepared from Huh-7 cells transfected with indicated plasmids. Ci and Xi denote expression cassettes of siRNA precursor targeting C and X ORF, respectively. Cm and Xm denote synonymous mutations in C and X ORF that confer resistance to Ci and Xi, respectively. Transfection efficiency was normalized using relative luciferase activities expressed by co-transfected firefly luciferase reporter measured using Luciferase Assay System (Promega); (**B**) ELISA analysis of HBsAg in culture media of infected PTH. Freshly prepared PTH were infected with wild type HBV and/or transfected Huh-7 cell supernatants as indicated. Media were changed and collected at indicated time points, and assayed for HBsAg; (**C**) Immunofluorescent analysis of intracellular HBcAg expression in infected PTH. Infected cells were fixed at 15 days post infection and intracellular HBcAg was detected using anti-HBcAg (Dako). Percentages of HBcAg-positive cells were calculated from ten randomly selected views and listed on the right. Cell nuclei were stained using DAPI. Scale bars, 200 µm.

We then went on to test the usability of 5dCG vector as potential HBV-targeting therapeutics. Expression cassettes of short interfering RNA (siRNA) precursors that were previously shown to target HBV C and X coding sequences using both plasmid and 5c3c vector [9] were amplified and cloned into 5dCG to create 5dCG-Ci and 5dCG-Xi respectively. Similar to parental 5c3c [9], replication efficiency of 5dCG-Ci and 5dCG-Xi in the presence of *trans-*complemented wild type C was significantly reduced compared to parental 5dCG, with replication of 5dCG-Ci nearly undetectable (Figure 7A). Targeting of both 5dCG-Ci pregenomic RNA and *trans-*complemented wild type C mRNA by Ci was partially responsible for reduced replication of 5dCG-Ci, because mutating the Ci target sequences in 5dCG-Ci to create 5dCG-Cm-Ci resulted in low but detectable replication, whereas mutating the helper plasmid pC to create pCm failed to support detectable replication from 5dCG-Ci (Figure 7A). 5dCG-Cm-Ci replicated more efficiently with co-transfected pCm than with wild type pC, but the efficiency was still inferior compared to 5dCG (Figure 7A). Similarly, when Xi target sequences in 5dCG-Xi were synonymously mutated to create Xi-resistant 5dCG-Xm-Xi, replication efficiency increased compared to 5dCG-Xi, but was still below the level of 5dCG (Figure 7A).

5dCG-Cm-Ci and 5dCG-Xm-Xi viruses were then tested for their effects on co-infecting wild type HBV in PTH infection assays. When HBsAg in PTH culture media was monitored over time as in Figure 6A (bottom), a similar pattern of decreasing background HBsAg levels was observed in PTH infected or co-infected with wild type HBV, with increased HBsAg secretion at day 6–8 p.i. (Figure 7B). Despite their relatively low replication efficiency (Figure 7A), 5dCG-Cm-Ci and 5dCG-Xm-Xi viruses were able to inhibit such HBsAg expression by co-infecting wild type HBV (Figure 7B). Similar inhibition of HBcAg expression by co-infecting wild type HBV was observed at day 15 p.i. (Figure 7C). Since 5dCG-sNLuc viruses had no such inhibitory effects (Figure 7B,C, also Figure 6A, bottom), inhibition through competition for receptor or other factors essential for HBV infection could be ruled out, and siRNA-mediated interference of wild type HBV translation and replication is most likely the underlying mechanism. These data demonstrated that 5dCG vectors delivering HBV-inhibiting cargo genes are indeed valid therapeutic candidates for HBV infection.

## 4. Discussion

Although so far limited to laboratory experiments, recombinant HBV has potentially widespread applications both as investigative tools *in vitro* and as therapeutic measures *in vivo*, owing to the extremely restricted species and tissue tropism of HBV infection and replication. In this work, we modified the cargo gene insertion strategy of our previously established highly replicative 5c3c recombinant HBV vector, in order to solve issues with 5c3c that affect its usability [9]. By moving the insertion site from preS/polymerase spacer region to core-coding region (Figure 1), we abolished the restriction imposed on cargo sequences in 5c3c that absolutely dictates the absence of terminator codon in the overlapping polymerase reading frame. Furthermore, introduction of a short artificial IRES upstream of polymerase start codon isolated upstream cargo sequences from downstream polymerase translation and improved replication competence (Figure 2B).

Replication efficiency was an issue with a majority of previously reported recombinant HBV designs [5,7,8,20]. Our previously described vector 5c3c was based on a highly replicative clinical isolate 6898, which harbors an in-frame deletion in preS/polymerase spacer region [9]. In transfection assays, both 6898 and 5c3c displayed replication efficiencies better than wild type HBV (Figure 3B) [9]. Presumably, shortened polymerase spacer plus decreased genome size conferred the replication advantage of 6898 and 5c3c. For this reason, the new vector 5dCG retained the maximized in-frame deletion in preS/polymerase spacer region in 5c3c and replication assay in transfected cells indeed showed that 5dCG vectors replicated much more efficiently than similar vectors derived from wild type HBV with full-length polymerase (Figure 2A). Retaining this deletion also has the added benefit of freeing up genome spaces for cargo gene insertions. These results reiterated the superiority of 5c3c and 5dCG vectors over previously reported recombinant HBV designs with regard to replication efficiency.

At least two categories of liver-targeting *in vivo* applications could be envisioned for recombinant HBV viruses. First, recombinant HBV expressing functional protein or RNA could be used to rectify a potentially life-long pathological condition, e.g., insulin for type I diabetes or coagulation factors for hemophilia [2]. In such cases, a self-replicating vector like 5c3c that, theoretically, replicates and maintains itself in infected hepatocytes for prolonged periods might be desirable. Another line of applications would involve using recombinant HBV expressing siRNA or miRNA precursors targeting wild type HBV, or immunomodulatory cytokines such as interferon α, as an alternative or supplementary treatment for chronic hepatitis B. In such cases, however, long-lasting persistence and activity of recombinant HBV after wild type HBV infection has been controlled would be undesirable, and may be justifiably considered a safety issue.

5dCG-based recombinant HBV does not encode functional core or envelope proteins, and would be entirely dependent on *trans-*complementation of these viral proteins from co-infecting or super-infecting wild type HBV *in vivo* for genome replication and virion production (Figure 2). Cargo gene expression apparently is also largely dependent on such *trans-*complementation (Figure 6A). In other words, HBV-targeting recombinant viruses, such as 5dCG-Cm-Ci and 5dCG-Xm-Xi, would replicate, expand and effectively produce HBV-targeting cargo gene products, as well as progeny viruses, when and only when there is significant co-existing wild type HBV activity in the same cell. In the absence of such *trans-*complementation, lack of self-sufficient replication would render these vectors generally inactive in infected hepatocytes (Figure 6A). Such “activated-by-target” qualities of 5dCG make it an ideal candidate for HBV-targeting therapeutic applications *in vivo*.

Before any human application could be attempted, recombinant HBV would need to be thoroughly studied for activity and long-term persistence in animal models. Unfortunately for HBV, up to now, this would have to rely on either chimpanzees or immuno-deficient mice harboring human hepatocytes, both of which are too expensive to allow studies involving large numbers of animals. Therefore, reporter viruses enabling non-invasive detection of recombinant HBV infection and expression would be invaluable in such cases. For replicating vector 5c3c, we reported the construction of 5c3c-DsRed virus [9], which could potentially allow detection of infected hepatocytes using *in vivo* fluorescence imaging. Similarly, for the non-self-replicating vector 5dCG, we constructed recombinant viruses expressing fluorescent proteins DsRed and EGFP. We also constructed 5dCG-sNL expressing a secreted form of NanoLuc luciferase, which would enable detecting recombinant HBV infection and activity by directly analyzing serum of infected animals. Furthermore, since 5dCG imposes no particular restriction on cargo gene sequences, other reporters allowing more convenient and more sensitive detection and measurement could be easily tested using 5dCG.

## 5. Conclusions

A previously established novel recombinant HBV vector 5c3c with high replication efficiency has been further improved by using a different cargo gene insertion site and insertion strategy. The resultant vector 5dCG allowed much freer choice of cargo sequences compared to 5c3c, while retaining 5c3c’s characteristic high replication efficiency. In addition, deletion of core-encoding sequences in 5dCG makes the vector totally dependent on wild type HBV for genome replication and progeny virus production, thus enhancing its safety for certain *in vivo* applications. The ability of 5dCG to deliver protein expression that allows non-invasive and non-disruptive detection of recombinant HBV activity, and its ability to deliver RNA interference to inhibit co-infecting wild type HBV were demonstrated using PTH model *in vitro*. The availability of recombinant HBV vectors 5c3c and 5dCG will no doubt inspire attempts at various liver-targeting human applications, whereas reporter viruses such as 5dCG-sNL will most certainly prove useful in preclinical studies of these applications.

## Supplementary Materials

The following are available online at www.mdpi.com/1999-4915/8/5/129/s1, Figure S1: Listing of partial sequences of 5dCG rHBV vector used in this work, Figure S2: Sendimentation analysis of wild type and recombinant HBV virions.
