# Supplementary material for: Re-Designed Recombinant Hepatitis B Virus Vectors Enable Efficient Delivery of Versatile Cargo Genes to Hepatocytes with Improved Safety
Source: Viruses. 2016 May 10;8(5):129. doi: 10.3390/v8050129 (PMC4885084; doi:10.3390/v8050129)
Supplement: Supplementary file 1 [file viruses-08-00129-s001.pdf]

Viruses **2016**, *8*, 129; doi:10.3390/v8050129 www.mdpi.com/journal/viruses

Supplementary Materials: Re-designed Recombinant Hepatitis B Virus Vectors Enable Efficient Delivery of Versatile Cargo Genes to Hepatocytes with Improved Safety

Weiya Bai, Xiaoxian Cui, Ruidong Chen, Shuai Tao, Ran Hong, Jiming Zhang, Junqi Zhang, Yongxiang Wang, Youhua Xie and Jing Liu

```
                                 5dCG

       M   Q   L   F   H   L   C   L   I   I   S   C   S   C   P
(1814)ATG CAA CTT TTT CAC CTC TGC CTA ATC ATC TCT TGT TCA TGT CCT
   preC start

 T   V   Q   A   S   K   L   C   L   G   W   L   W   G   M
ACT GTT CAA GCC TCC AAG CTG TGC CTT GGG TGG CTT TGG GGC ATG
                                                        C start

 D   I   D   T   Y   K   E   F   G   A   S   V   E   L   L
GAC ATT GAC ACG TAT AAA GAA TTT GGA GCT TCT GTG GAG TTA CTC

 S   F   L   P   S   D   F   F   P   S   I   Q   D   L   I
TCT TTT TTG CCT TCT GAC TTC TTT CCT TCT ATT CAA GAT CTC ATC

 D   T   A   S   A   *      PstI          NheI
GAC ACC GCC TCT GCT TAA CCC TGC AGG CTC GCT AGC CTG TTG CCC

TCT GGT TTC TCC CCA GGT TAT CCG GCG GGT TTC TGA CAT CCG GCG
                       Green: Gtx IRES

                                             M   P   L   S   Y   Q .
GGT GAC TCA CAA CCC CAG AAA CAG ACA TAT GCC CCT ATC TTA TCA
                                         P Start

  H   F   R   K   L   L   L   L   D   D
ACA CTT CCG GAA ACT ACT GTT GTT AGA CGA C(2354)
```

Figure S1. Listing of partial sequences of 5dCG rHBV vector used in this work. Sequences surrounding the cargo sequence insertion site are shown. Numbers indicate nucleotide positions on wild type HBV genome. Start codons of preC, C and P ORF as well as artificially introduced premature termination codon (*) of preC/C ORF are indicated. Gtx IRES sequences introduced upstream of P start codon are highlighted in green. Restriction enzyme recognition sites used for inserting cargo sequences are also shown.

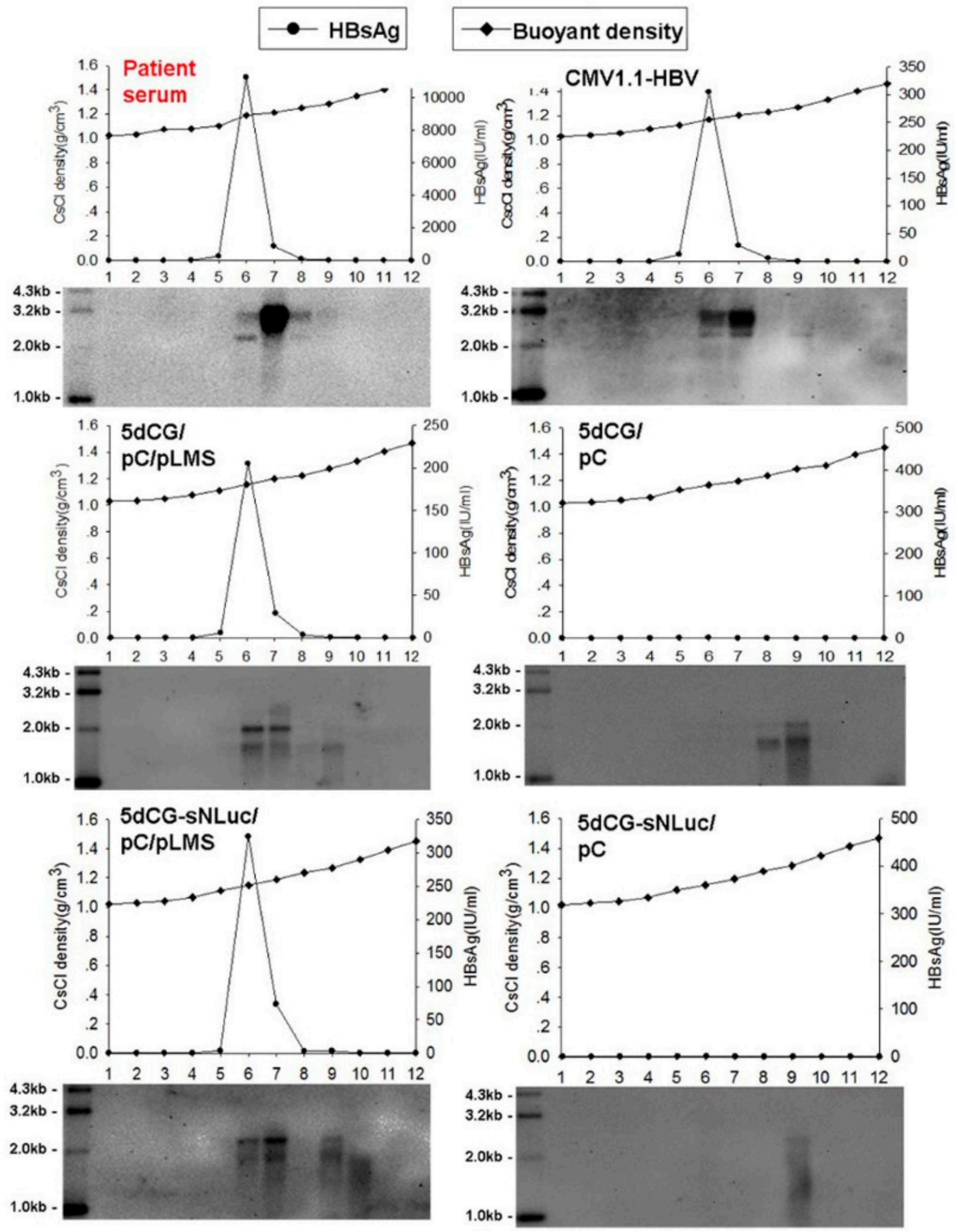

Figure S2. Sendimentation analysis of wild type and recombinant HBV virions. Culture supernatants of Huh-7 cells transfected with indicated plasmids, as well as HBV-infected patient serum, were subjected to CsCl density gradient ultracentrifugation. Fractions were analyzed for CsCl density (solid diamonds), HBsAg (solid circles) and HBV DNA.

© 2016 by the authors; licensee MDPI, Basel, Switzerland. This article is an open access article distributed under the terms and conditions of the Creative Commons by Attribution (CC-BY) license (http://creativecommons.org/licenses/by/4.0/).
